# pH testing to confirm nasogastric tube position on the ICU: are we wasting our time?

**DOI:** 10.1186/cc12183

**Published:** 2013-03-19

**Authors:** P Temblett, S George

**Affiliations:** 1 Morriston ICU, Swansea, UK

## Introduction

For such a simple procedure, the insertion and position checking of nasogastric (NG) tubes can be very problematic. The UK's National Patient Safety Agency declared that 'Placement of NG tubes together with confirmation of correct placement can carry significant risks' and recommends that measuring the pH of NG aspirate should be the first-line method of determining correct NG position (safe range ≤5.5) [1].

## Methods

In order to assess the usefulness of pH testing of NG aspirates in critical care patients, a prospective survey of pH testing of NG tube aspirate was carried out. This was undertaken both in patients who had a newly placed NG tube and in patients who were having regular/ routine checks of their existing NG tube.

## Results

**Figure 1:**
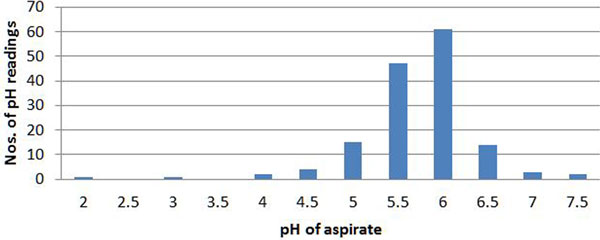
**pH values - routine checks**.

**Figure 2:**
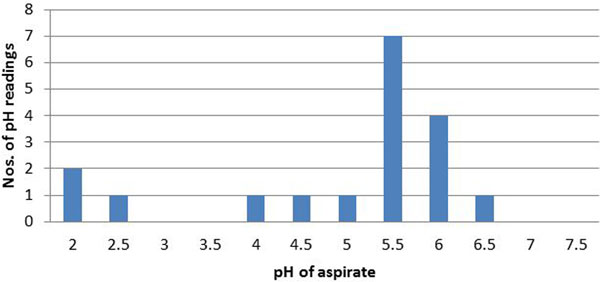
**pH values - new NG tubes**.

A total of 168 separate pH readings in 41 ICU patients receiving continuous enteral nutrition were recorded. In total, 137/168 patients were receiving proton pump inhibitors (PPIs). Eighteen readings were from newly placed NG tubes and 150 readings from old NG tubes. Fifty-three per cent of routine pH readings were falsely high; that is, pH 6 or above despite the NG tube being in the stomach (Figure 1). Twenty-eight per cent of newly placed NG tubes had falsely high pH readings (Figure 2).

## Conclusion

In this population of ICU patients, routine/daily checks of NG pH aspirate appear to be limited. This is almost certainly due to the use of continuous NG feed together with PPIs. The usefulness of pH testing in newly placed NG tubes, however, appears more reliable.
